# A genome-wide association study of folates in sweet corn kernels

**DOI:** 10.3389/fpls.2022.1004455

**Published:** 2022-09-30

**Authors:** Yingni Xiao, Yongtao Yu, Lihua Xie, Kun Li, Xinbo Guo, Guangyu Li, Jianhua Liu, Gaoke Li, Jianguang Hu

**Affiliations:** ^1^ Crops Research Institute, Guangdong Academy of Agricultural Sciences/Guangdong Provincial Key Laboratory of Crop Genetic Improvement, Guangzhou, China; ^2^ School of Food Science and Engineering, South China University of Technology, Guangzhou, China

**Keywords:** sweet corn, folates, genome-wide association study, genetic basis, formyltetrahydrofolate cyclo-ligase

## Abstract

Folate is commonly synthesized in natural plants and is an essential water-soluble vitamin of great importance inhuman health. Although the key genes involved in folate biosynthesis and transformation pathways have been identified in plants, the genetic architecture of folate in sweet corn kernels remain largely unclear. In this study, an association panel of 295 inbred lines of sweet corn was constructed. Six folate derivatives were quantified in sweet corn kernels at 20 days after pollination and a total of 95 loci were identified for eight folate traits using a genome-wide association study. A peak GWAS signal revealed that natural variation in *ZmFCL*, encoding a 5-formyltetrahydrofolate cyclo-ligase, accounted for 30.12% of phenotypic variation in 5-FTHF content. Further analysis revealed that two adjacent SNPs on the second exon resulting in an AA-to-GG in the gene and an Asn-to-Gly change in the protein could be the causative variant influencing 5-FTHF content. Meanwhile, 5-FTHF content was negatively correlated with *ZmFCL* expression levels in the population. These results extend our knowledge regarding the genetic basis of folate and provide molecular markers for the optimization of folate levels in sweet corn kernels.

## Introduction

Folate or vitamin B9, is a generic term for tetrahydrofolate (THF) and its C_1_-subsituted derivatives, which are synthesized *de novo* in bacteria, fungi and plants. Folates serve as donors and acceptors in one-carbon (C1) transfer reactions, making them essential in all living organisms (Basset et al., 2005). An adult human should consume 400 mg folates per day and pregnant women should supplement their diet with 600 mg of folates per day (http://ods.od.nih.gov/factsheets/folate.asp). Unfortunately, the most important staple crops in folate production are poor suppliers of folates, hence, folate deficiency has become a significant public health problem, especially in developing countries (Strobbe and Van Der Straneten, 2017). Folate deficiency increases the risk of various diseases, such as cardiovascular diseases and certain types of cancers (Blancquaert et al., 2010; Herrera-Araujo, 2016). In addition to supply of folates in pill forms, biofortification of daily food is considered a convenient and safe approach to ameliorate folate deficiency (Bekaert et al., 2008).

The folate metabolism pathway has been fully elucidated by several reviews (**Supplementary Figure S1**) (Hanson and Gregory, 2011; Blancquaert et al., 2014; Gorelova et al., 2017). The THF molecule is composed of three moieties: a pterin ring, a para-aminobenzoate (pABA) and a glutamate tail (Gorelova et al., 2017). Pterin and pABA are synthesized in cells by several enzymes in the cytosol and plastids, respectively (Basset et al., 2002; Basset et al., 2004). Next, pterin and pABA are transported into the mitochondrion for the assembly of THF (Basset et al., 2005). Furthermore, during the transformation of THF to 5, 10-methylene THF, serine (Ser) is generated and serves as an alternate donor of C1 metabolism (Fox and Stover, 2008). According to the number of glutamate residues, which varies from 4 to 6, seven distinct folate species can be distinguished in plant, including THF, 5-MTHF, 5,10-CH2-THF, 5,10-CH=THF, 5-F-THF, 10-formyl THF and 10-formimino THF (Gorelova et al., 2017). Overall, there are dozens of enzymes that are involved in folate and C1 metabolism, and their corresponding genes are well conserved in plants (**Supplementary Figure S1**). Although in general the process of folate metabolism in plant is well understood, and the two key genes involved (*GTPCHI* and *ADCS*) have been successfully applied to enhance folate content in maize and other plants (Storozhenko et al., 2007; Naqvi et al., 2009; Liang et al., 2019), the regulation of folate metabolism pathway remains unclear.

In last ten years, genome-wide association studies (GWAS) have become a powerful tool to identify new genes associated with nutritional traits in corn (Li et al., 2013; Liu et al., 2016; Diepenbrock et al., 2017; Baseggio et al., 2020);. Recently, two major QTLs for 5-FTHF were detected using a segregated population in field corn kernels (Guo et al., 2019). As a special corn, sweet corn is distinguished from field corn by many genes and is harvested at the milk-ripe stage (Scott et al., 2019; Hu et al., 2021), its consumption rates have rapidly increased in China recently. As yet, little is known about the genetic architecture of folates in sweet corn kernels. In this study, an association population consisting of 295 inbred lines was constructed (Li et al., unpublished), genome-wide association analysis was performed for folate traits. Our objectives were (1) to generate a comprehensive understanding of the folate content of sweet corn kernels, (2) to identify novel genes that contribute to folate levels and (3) to provide an informative platform for folate content improvement *via* molecular breeding in sweet corn.

## Materials and methods

### Plant material and sampling

In a previous study, we constructed a diverse natural population of 204 sweet corn inbred lines and successfully dissected the genetics of vitamin E in this population (Xiao et al., 2020). In the present study, we added further sweet corn inbred lines with different types, and constructed a new association panel including 295 sweet corn inbred lines. The population was planted in a randomized complete block design with three replications at Handan, Hebei Province of China in the summer of 2017. At the seedling stage, bulks leaf samples of each of the 295 inbred lines were obtained from five individual plants in each line for genomic DNA extraction. All ears in each block were self-pollinated, and six immature kernels from three ears of each line in one replicate were collected 15 days after pollination (DAP) for total RNA extraction. Additionally, for each line in three replicates, intact immature kernels were separated from three ears at 20 DAP and frozen in liquid nitrogen for folate measurement.

### Whole genome resequencing and RNA sequencing

Genomic DNA of accessions were extracted using the CTAB protocol (Murray and Thompson, 1980) before being sequenced using the Illumina HiSeq 2500 platform, yielding 7.3 Tb of data with an average depth of 11.8-fold (ranging from 10- to 12-fold) (Li et al., unpublished). The reads was first controlled with Trimmomatic 3.0 (Bolger et al., 2014) and then mapped against the B73 reference genome (RefGen_V4) using BWA software. Samtools was used to remove reads with a mapping quality (MAPQ) lower than 30. GTAK (version 4.1.3) was used to call each SNP using the best-practice pipeline (Mckenna et al., 2010). Further, we removed SNPs with a missing ratio > 10% or MAF < 5%. Finally, a total of 9.86 million high-quality SNPs were generated, covering about 77% of annotated maize genes.

Total RNA was extracted for RNA sequencing using a Quick RNA Isolation Kit. Libraries were constructed with a 300 to 500 -bp insert size, and 150-bp paired-end Illumina sequencing. A total of 31.27 billion raw reads per sample were obtained. Reads were mapped to the B73 reference genome (RefGen_V4) using Hisat2 (Pertea et al., 2016). Next, StringTie (Pertea et al., 2016) was used to assemble transcripts and estimate their expression abundancies. FPKM values were calculated for all genes in each sample using the Ballgown package in R software (version 3.6.0) (Lhaka and Gentleman, 1996). Finally, an expression profile of 27,133 genes in the whole genome for all lines was obtained (Li et al., unpublished).

### Determination of folate levels and statistical analysis

Folate extraction and detection was carried out as previously reported (Liang et al., 2019). In brief, 10 fresh kernels from each sample were ground into powder in liquid nitrogen. Next, 0.05g of powder was added to extraction buffer and rat serum, successively. The folate extraction was determined by chromatographic analysis with an Agilent 1260 HPLC system (Palo Alto, CA). Six folates were determined within the population, including 5,10-methenyltetrahydrofolate (5,10−CH=THF), 5-formyltetrahydrofolate (5-FTHF), 5-methyltetrahydrofolate (5-MTHF), dihydrofolate (DHF), tetrahydrofolate (THF) and folic acid. According to the pathway, we calculated three secondary traits, including total folate, the ratio of 5,10−CH=THF/5-FTHF and the ratio of DHF/THF.

For each line, the best linear unbiased predictor (BLUP) was calculated using the *lme4* package of R as follows: *y_i_* = *μ* + *g_i_* + *e_i_* + *ϵ_i_*, where µ is the mean, *g_i_* represents the genetic effect, *e_i_* is the environmental effect, and *ϵ_i_* is denoted as the residual error. The broad-sense heritabilities (*h^2^*) of each traits was estimated as *h^2^* = *σ_g_^2^*/(*σ_g_^2^* + *σ_ϵ_^2^*/*e*), where *σ_g_^2^* represents the genetic variance, and *σ_ϵ_^2^* represents the residual error, and *e* is the number of environments (Knapp et al., 1985).

### Genome-wide association analysis

Relative kinship analysis was performed using the Centered-Identity by State (Centered-IBS) matrix values in TASSEL (v.3.0) (Bradbury et al., 2007). The population structure was estimated by PLINK (v.1.90) (Chang et al., 2015) and the top five principle components (PCs) were selected to represent the population structure of sweet corn inbred lines. The genome-wide average *r^2^* between two SNPs within 600-kb windows was calculated by PopLDdecay (v3.40). Overall, with the threshold set to *r^2 =^* 0.2, the distance of LD decay was 35 kb in the population. A GWAS regarding folates traits was conducted in TASSEL (v.5.0) using a mixed linear model by control of both population structure and kinship. For conditional GWAS, the genotype of the leading site was extracted and combined with covariates to detect additional significant sites. Considering the strong LD among all SNPs, a total of 767964 independent SNPs were determined by PLINK (window size 50, step size 50, *r^2^* ≥ 0.2) (Purcell et al., 2007) and thus, a Bonferroni-adjusted significance threshold (P < 1.0 × 10^-6^ = 1/767964) which followed the significance level similar to a previous study (Li et al., 2013) was used to identify significant associations. Adjacent significant loci (< 500 kb) were treated as one leading SNP based on LD statistics (*r^2^* ≥ 0.2). For each trait, the phenotypic variation of the population explained by all significant leading SNPs was estimated by stepwise regression, using the *lm* function in R (Li et al., 2013).

All genes within 35 kb up- and downstream of the leading SNPs were considered as acceptable associated genes, and the gene in or near each peak which were expressed in kernels at 15 DAP (FPKM > 0 in more than one line of the sweet corn population) were proposed to be the most likely candidate for association.

### *ZmFCL*-based association analysis

According to the sequences of B73 and RC (an inbred of sweet corn, Li et al., unpublished), two pairs of primers were designed to amplify *ZmFCL* from 215 sweet corn inbred genotypes (**Supplementary Table S1**). The sequences were aligned and refined manually in BioEdit (Hall, 1999). DNA variations, including SNPs and indels were extracted, then the *r^2^* among polymorphisms were calculated by TASSEL. The gene-based association analysis was conducted in TASSEL (v.5.0) using a mixed linear model by control of both population structure and kinship as previously mentioned.

## Results

### Phenotypic variation of folates in sweet corn kernels

**Figure 1:**
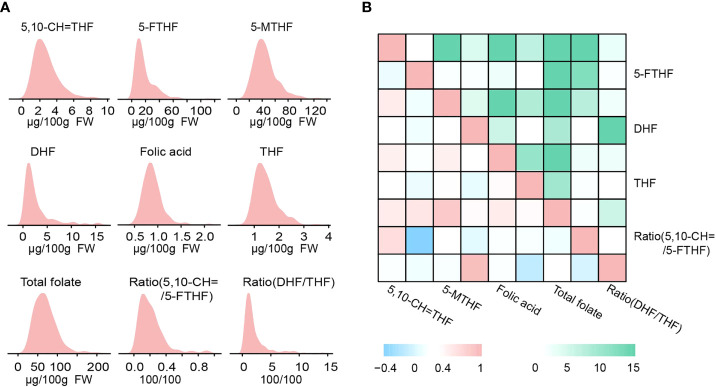
Phenotypic variation of nine folate traits in this study. **(A)** Phenotypic distribution of nine folate traits in the population. **(B)** Pearson correlation coefficients (bottom left) for the nine traits and –log_10_ (*P*-value) of the Pearson correlation (upper right).

In sweet corn kernels, the levels of six folate derivatives including 5,10−CH=THF, 5-FTHF, 5-MTHF, DHF, THF and folic acid were quantified by HPLC-MS/MS. Among them, 5-MTHF was the most abundant derivative, followed by 5-FTHF, accounting for 66.0% and 26.2% of total folates, respectively. In contrast, folic acid was the least abundant (**Table 1**, **Figure 1A**). Furthermore, the sweet corn population analyzed herein exhibited large variation in nine folate traits, with a range of variation of 4.4 to 38.1 fold (**Table 1**, **Figure 1A**). Most of the traits exhibited continuous and approximately normal distribution (**Figure 1A**). A strong negative correlation was observed between 5-FTHF and the Ratio of (5,10−CH=THF/5-FTHF) (*r*=-0.46, *P*=3.61×10^-12^), while positive correlations were observed among most traits (**Figure 1B**). An analysis of variance (ANOVA) revealed that genotype variance was greater than environmental variance in all traits, and their broad-sense heritability ranged from 0.65 to 0.98 (**Table 1**), indicating that the phenotypic variations were mainly controlled by genetic factors. Thus, the abundant phenotypic variation and high heritability of nine folate traits in the population provide a genetic basis for identifying new sites in sweet corn.

**Table 1:** Statistical summary, broad-sense heritability and variances of folates in the sweet corn population.

Trait	Number of lines	Range	Mean ± SD	*h^2a^*	Variance^b^		
					*σ_g_^2^*	*σ_e_^2^*	*σ*_ϵ_ *^2^*
5,10-CH=THF(μg/100g FW)	245	0.60-8.49	2.72 ± 1.41	0.82	1.77**	0.06**	1.13
5-FTHF(μg/100g FW)	233	3.57-111.05	18.59 ± 14.49	0.98	148.20**	0.14**	8.43
5-MTHF(μg/100g FW)	247	14.56-124.62	44.67 ± 17.32	0.79	296.57**	-0.06	235.43
DHF(μg/100g FW)	241	0.15-15.72	2.76 ± 2.97	0.95	6.35**	0.07**	1.1
Folic acid(μg/100g FW)	248	0.48-2.10	0.88 ± 0.20	0.65	0.05**	4.14×10^-6^	0.08
THF(μg/100g FW)	247	0.69-3.50	1.46 ± 0.44	0.71	0.21**	0.007**	0.25
Total folate(μg/100g FW)	219	21.64-198.22	69.43 ± 27.73	0.86	488.18**	1.13	235.6
Ratio(5,10-CH=THF/5-FTHF)	231	0.04-0.92	0.22 ± 0.16	0.86	0.02**	4.15×10^-4^**	0.01
Ratio(DHF/THF)	240	0.37-14.08	2.08 ± 2.04	0.86	3.09**	0.05**	1.5

^a^Broad-sense heritability of nine folates traits in the population.

^b^σ_g_^2^ is genetic variance, σ_e_^2^ is the environment variance and σ_ϵ_^2^ is the residual variance. *P < 0.05, **P < 0.01.

### GWAS of folate traits in sweet corn kernels

**Figure 2:**
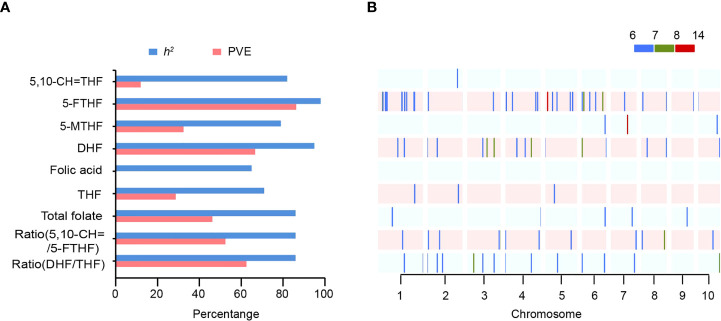
Summary of significant sites for folate traits identified by GWAS. **(A)** Broad-sense heritability (*h^2^*) and total PVE for each folate trait in the population. **(B)** Distribution of significant sites on chromosomes. Regions across the maize genome are represented within 35 kb of the most significant site intervals, and –log_10_ (*P*-value) are scaled by color.

Using 9.86 million SNPs from 295 lines, we performed a genome-wide association study (GWAS) to identify the novel sites for each of the nine folates in sweet corn kernels. A total of 96 sites were significantly associated with eight of the folates at a threshold of P<1.0×10^-6^ (**Figure 2**; **Table 2**; **Supplementary Table S2**; **Supplementary Figure S2**). For these eight traits, the phenotypic variation explained by each SNP ranged from 7.55% to 30.12%, with the lowest and highest phenotypic variation both detected associated with 5-FTHF. Thirty-four SNPs were significantly associated with 5-FTHF, exhibiting the greatest phenotypic variation (86.33%, **Figure 2**; **Table 2**). In contrast, one SNP was associated with 5,10-CH=THF, exhibiting the least phenotypic variation (11.94%). Among all the SNPs, nine sites, including S1_177585636, S2_370059, S2_4133879, S2_69899086, S3_110458943, S3_186220703, S4_179431728, S6_7251161 and S10_149171639, were significantly associated with both DHF and the DHF/THF ratio, while one site (S6_160666250) was significantly associated with both 5-MTHF and total folate (**Supplementary Table S2**), exhibiting pleiotropic effects.

**Table 2:** Summary of significant SNPs identified for folates traits in sweet corn.

Trait	Significant SNPs	Variation explained by each SNP (%)	Variation explained by all SNP (%)
5,10-CH=THF(μg/100g FW)	1	11.94	11.94
5-FTHF(μg/100g FW)	34	7.55-30.12	86.33
5-MTHF(μg/100g FW)	3	12.35-16.65	32.43
DHF(μg/100g FW)	18	11.84-18.25	66.67
Folic acid(μg/100g FW)	0	0	0
THF(μg/100g FW)	3	11.37-13.11	28.66
Total folate(μg/100g FW)	6	12.50-13.85	46.21
Ratio(5,10-CH=THF/5-FTHF)	13	11.89-15.72	52.43
Ratio(DHF/THF)	18	12.14-15.76	62.59

According to annotation of the B73 genome (RefGen_V4) and analysis of the expression data from immature sweet corn kernels at 15 DAP, a total of seventy-three genes were considered to be potential candidate genes (**Supplementary Table S2**). Gene set enrichment analysis revealed significantly enriched terms (*P*<0.05) in sphingolipid metabolism, circadian rhythm and cysteine and methionine metabolism (**Supplementary Figure S3**), indicating that several candidate genes identified were involved in lipid and amino acid metabolism. In addition, several transcription factors were identified associated with folates in sweet corn kernels (**Supplementary Table S2**). As the second most abundant folate derivative in sweet corn kernels, a series of strong signal sites on chromosome 5 were associated with 5-FTHF levels (**Supplementary Figure S2**). The most significant site (S5_20162982), accounted for 30.12% of the phenotypic variation, was located on the second exon of Zm00001d013786 (**Supplementary Table S2**). However, S5_20162982 was a synonymous SNP that resulted no amino acid change within the coding region of the gene. Zm00001d013786 encoded a 5-formyltetrahydrofolate cyclo-ligase protein, which catalyzes the conversion of 5-FTHF into 5,10-CH=THF; the final step of the folate transformation pathway (**Supplementary Figure S1**), and the gene is herein assigned as *ZmFCL*. Thus, *ZmFCL* is a potential candidate gene for 5-FTHF content in sweet corn kernels.

### *ZmFCL* is significantly associated with 5-FTHF levels

**Figure 3:**
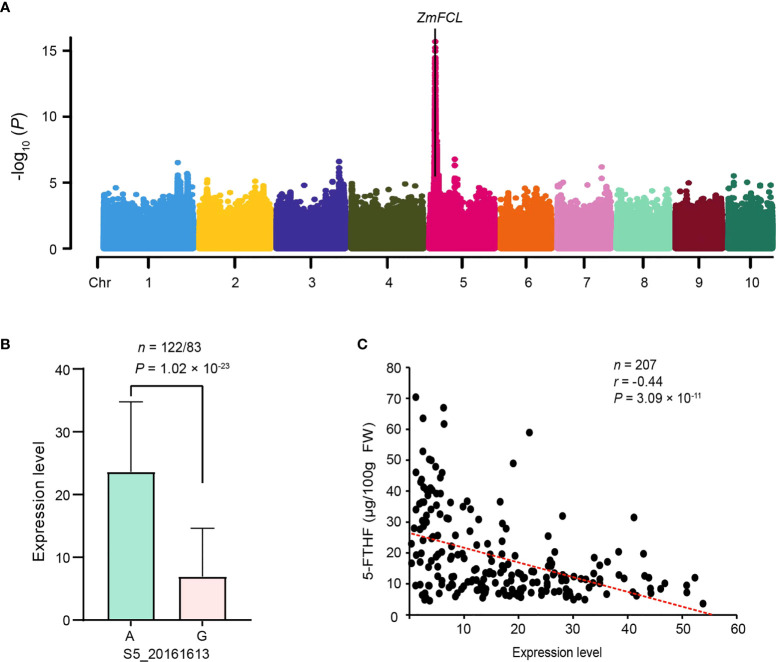
Expression analysis of *ZmFCL*. **(A)** Manhattan plot for the expression GWAS of *ZmFCL* with eQTL. **(B)** Comparison of *ZmFCL* expression between different alleles of the significant SNP (S5_20161613). The *P* value is based on a two-tailed *t*-test. *n* denotes the number of genotypes belonging to each allele group. **(C)** The correlation between the expression level of *ZmFCL* and 5-FTHF. The *x* axis represents the expression of *ZmFCL* in kernels collected at 15 DAP. The *y* axis represents the 5-FTHF. *n* denotes the number of inbred lines of sweet corn. The *r* value is a Pearson correlation coefficient.

To gain a preliminary understanding of the regulation *ZmFCL*, its expression level in the population was treated as the phenotypic variable and GWAS was performed to explore its eQTL. The site (S5_20161613) significantly associated with its expression level was located in the promoter region of *ZmFCL* (**Figure 3A**), implying that *ZmFCL* was a cis- eQTL. The sweet corn inbred lines with the G allele showed significantly lower expression levels than lines with the A allele at the S5_20161613 site (**Figure 3B**). Moreover, subsequent investigation revealed that the expression of *ZmFCL* was strongly negatively correlated with 5-FTHF content (*r* = -0.44, *P* = 3.09 ×10^-11^) (**Figure 3C**). The pattern of expression level in the population was consistent with the biochemical function of the enzyme encoded by *ZmFCL*, which converts 5-FTHF to 5,10-CH=THF (**Supplementary Figure S1**).

**Figure 4:**
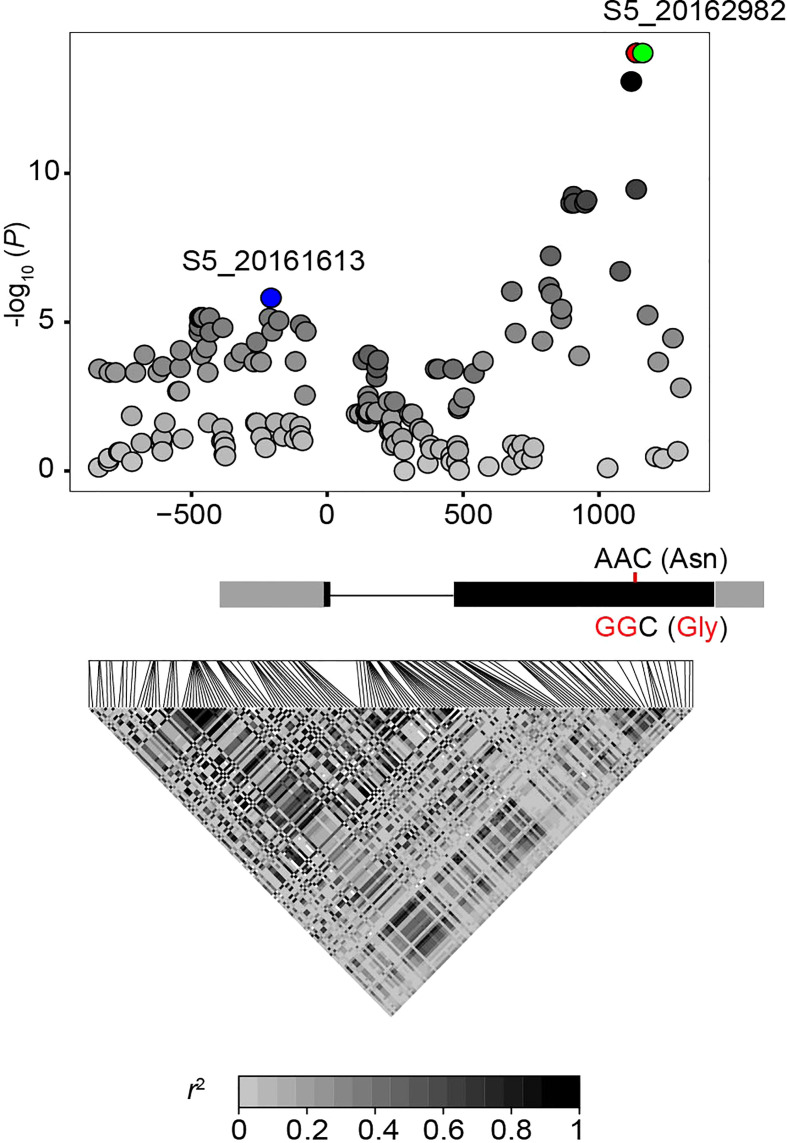
*ZmFCL*-based association mapping and LD analysis of 215 sweet corn inbred lines. The most significant SNP (S5_20162982) of GWAS for 5-FTHF is indicated in green, while the most significant SNP of *ZmFCL* expression is indicated in blue. The two adjacent significant SNPs were highlighted with red dots. The intensity of gray shading indicates the extent of linkage disequilibrium (*r^2^*) between the leading SNP (S5_20162982) and the other variants identified in this region. The gene structure is shown below the x axis. Black and grey boxes represent exons and UTRs, respectively. The red nucleotides indicate nonsynonymous SNP substitution in the second exon of *ZmFCL*.

To further determine the potential causative variant of *ZmFCL*, we re-sequenced *ZmFCL* in 215 sweet corn inbred lines. Sequence analysis revealed that 163 variants in the promoter region and the coding region of full-length *ZmFCL* (**Figure 4**). Of these 163 variants, 56 were significantly associated with 5-FTHF content (*P* ≤ 3.07×10^-4^ = 0.05/163) (**Figure 4**). Three SNPs exhibited the strongest association with 5-FTHF content (*P* = 8.93 × 10^-15^, *n* = 205), including the S5_20162982 site detected by the GWAS. Two additional SNPs were located 22-bp and 21-bp away from S5_20162982, revealing complete linkage with S5_20162982 (*r^2^* = 1). Unlike S5_20162982, these two SNPs were nonsynonymous with an AA-to-GG change resulting in an Asn-to-Gly change (**Figure 4**), implying a potential functional site within *ZmFCL*. In addition, several sites in the 5’UTR and promoter regions of *ZmFCL* were significantly associated with 5-FTHF levels due to strong linkage with the identified functional site (*r^2^* > 0.5) (**Figure 4**).

### Haplotype analysis of *ZmFCL* revealed variable effects on 5-FTHF content

**Figure 5:**
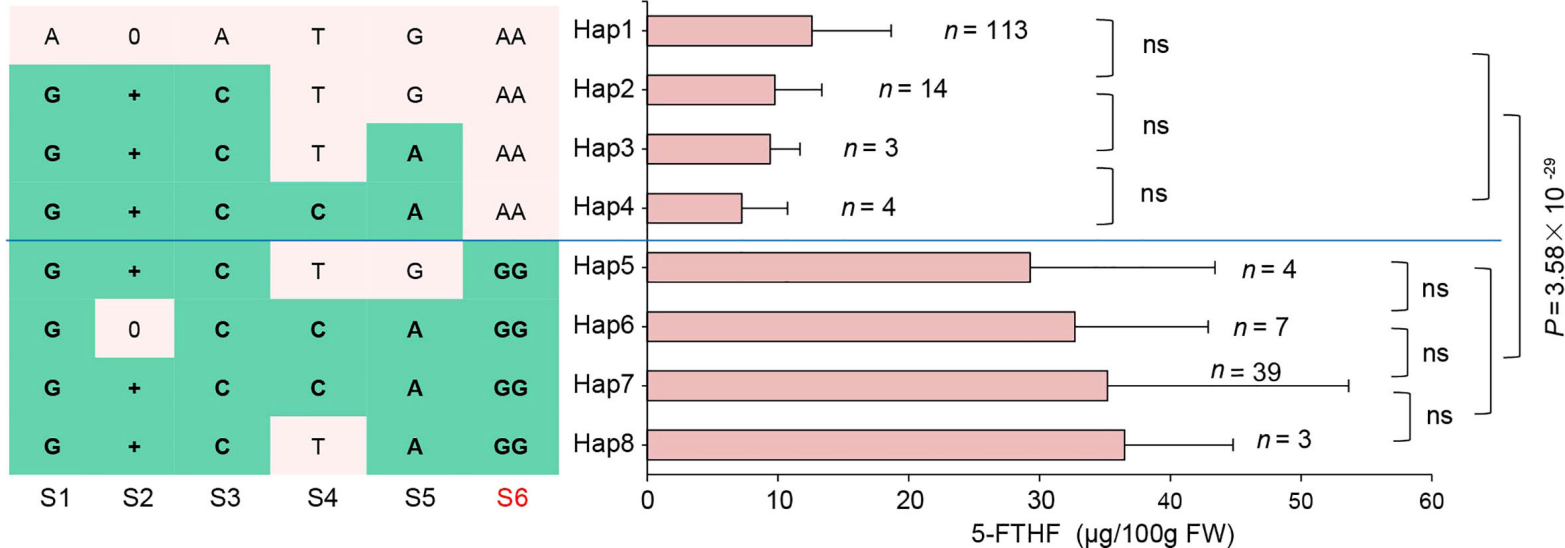
Haplotypes of *ZmFCL* among natural variations in sweet corn inbred lines. *n* denotes the number of genotypes belonging to each haplotype group. When a string of variations are in complete LD, only one is shown. S6 was the causative variant mentioned above. Statistical significance was determined by a two-sided t-test. *ns* denotes no significance between two groups.

To estimate the effects of haplotypes on 5-FTHF content in the sweet corn panel, the top six significant variants were extracted based on candidate gene association analysis. The S1 site was the SNP (S5_20161613) that was significantly associated with *ZmFCL* expression, located within the promoter region of *ZmFCL*. The additional variants were all located in the second exon. The site (S2) was an insertion with 3/9/12-bp in the second exon, resulting in a 1/3/4 amino acid insertion in the protein. The additional sites (S3, S4 and S5) were all synonymous, while the final site (S6) was the functional site causing an Asn> Gly change mentioned previously. A total of 13 haplotypes were detected in 193 inbred lines. Eight of these haplotypes had a sample size of > 2 lines (frequencies > 0.01) (**Figure 5**). The eight haplotypes had heterogeneous effects on 5-FTHF content. As with the majority of test lines, Hap1 (*n* = 113) resulted in lower 5-FTHF content, showing no significant disparity with Hap2, Hap3 and Hap4. In contrast, Hap5, Hap6, Hap7 and Hap8 led to higher 5-FTHF content, showing no significant differences amongst each other. When GG haplotypes (Hap5-8) were compared with AA haplotypes (Hap1-4), a highly significant difference in 5-FTHF level was observed (P = 3.58 × 10^-29^, *n* = 193) (**Figure 5**). These results further demonstrated that the two SNPs (AA -to-GG) represented the causative site of *ZmFCL* for 5-FTHF content. Additionally, the favorable allele (GG) was present in about one third of total inbred lines in the sweet corn panel (**Figure 5**), implying the great potential for application in sweet corn.

## Discussion

### Folate profiling in sweet corn kernels

In the study presented herein, a panel consisting of 295 sweet corn inbred lines collected from different habitats was constructed for analysis of folate concentrations. 5-MTHF was the most abundant folate derivative identified, followed by 5-FTHF, in agreement with that folate pool *in- vivo* is largely dominated by 5-MTHF and 5-FTHF (Cossins, 2000). Similarly, 5-MTHF was the dominate folate identified in early developing field corn kernels (Lian et al., 2015). Moreover, 5-FTHF was the most abundant folate derivative identified in field corn kernels (Guo et al., 2019), in agreement with that 5-FTHF was the most stable natural folate in seeds (Ravanel et al., 2011). In general, the folate composition in plants greatly fluctuates during the course of development, implying that folate regulation is modulated as a function of metabolic requirements (Jabrin et al., 2003). Therefore, the folate regulation mechanism in sweet corn kernels is distinct from in the dry seeds of field corn. In addition, we detected folic acid in sweet corn kernels, which was not detected in the dry seeds of field corn (Guo et al., 2019), implying the difference between sweet corn and field corn. Overall, our results represents a comprehensive folate profiling of sweet corn kernels.

### Genetic architecture and potential candidate genes for folates in sweet corn kernels

Nine traits associated with folate levels were determined in this study. Thirty-four sites were significantly associated with 5-FTHF, which exhibited the greatest level of heritability. In contrast, no sites were associated with folic acid, which showed the least heritability (**Figure 2**). The SNP on chromosome 5 contributed to the large phenotypic variation in 5-FTHF (*r^2^* >30%, **Figure 2**, **Supplementary Table S2**), in agreement a major QTL was previously detected in the same location in field corn (Guo et al., 2019). It was suggested that 5-FTHF is regulated by a major gene in addition to a multitude of minor genes. For the remaining traits, the phenotypic variation associated with each SNP ranged from 7.55% to 18.25%, suggesting that such traits were regulated by minor poly genes.

Dozens of genes were associated with folate levels in sweet corn kernels (**Supplementary Table S2**). The gene (Zm00001d038595), which encodes a myosin heavy chain-related protein, was significantly associated with 5-MTHF and total folate concentrations. Another gene (Zm00001d031532), which also encodes a myosin heavy chain-related protein, was significantly associated with 5-FTHF. Myosins, as molecular motor proteins, participate in the growth and development of plants (Madison and Nebenfuhr, 2013; Henn and Sadot, 2014). *O1* encodes a myosin XI protein that affects protein production and folding in maize kernel (Wang et al., 2012). According to this study, myosins may regulate folate generation in sweet corn kernels, suggesting distinct functional roles. The gene (Zm00001d035157), which encodes a lysine histidine transporter, was significantly associated with DHF levels and the DHF/THF ratio. The expression of this gene can be down-regulated by *ZmPHR1* transcription factors in maize in low phosphate conditions, leading to a decrease in grain numbers (Wang et al., 2021). However, the mechanism by which the gene Zm00001d035157 regulates DHF and the DHF/THF ratio should however be further validated by molecular biology analysis.

### The functional conservation of *ZmFCL* and its potential application

In this study, *ZmFCL* was a strong candidate gene associated with 5-FTHF content in sweet corn kernels. 5-FCL, a 326 amino acid protein transcribed from the *ZmFCL* gene, consists of two domains (N-terminal and C-terminal subdomain) (**Supplementary Figure S4A**). A phylogenetic tree exhibiting 14 proteins from *ZmFCL* orthologs was constructed by MEGA and the results demonstrated that the protein was highly conserved within monocots with the highest level of conservation between sweet corn and field corn (**Supplementary Figure S4B**). Two SNPs within the functional site of 5-FCL caused an amino acid substitution from Asn (N) to Gly (G) occurred in the 228^th^ of the amino acid which was predicted in the 7^th^ β-strand of the protein (**Supplementary Figure S5**) (Kelley et al., 2015). The 228^th^ amino acid which near the conserved region (amino acid YN) might result in an alteration to the activity of 5-FCL (**Supplementary Figure S5**). Furthermore, the amino acid substitution from “N” to “G” was predicted to be functional by PPVED (predicted score 0.91), consistent with our speculation. There are three amino acids (N/G/S) in the 228^th^ site among the eight monocot species. It is interesting that the amino acid substitution from “N” to “S” was predicted to be neutral (predicted score 0.34) by PPVED (Gou et al., 2022), implying that the “G” was the favorable amino acid compared to “N” and “S”. The molecular mechanism of the functional site identified herein requires further validation.

Natural folate derivatives in plants are an important source of folates for humans and biofortification of plant food sources would be a cost-effective complementary strategy to fight folate deficiency in the developing world (De Steur et al., 2015). Genome-wide association studies (GWAS) combined with marker assisted breeding have been shown to be a powerful tool for biofortification of nutritional traits in corn (Yan et al., 2010; Yang et al., 2018; Xiao et al., 2020). As the major derivative in both sweet corn and field corn kernels, 5-FTHF was an ideal compound to target for biofortification. Surprisingly, the favorable allele of *ZmFCL* was present in about one third of total germplasm in the association panel (**Figure 5**), implying that the favorable alleles already exist in sweet corn germplasm throughout the world. It is feasible to enhance 5-FTHF content in a wide range of genetic backgrounds using marker assisted selection. Furthermore, the unfavorable allele of *ZmFCL* was present in most of the major crops, such as, field corn and rice (**Supplementary Figure S5**), suggesting the great potential application on other crops. Additionally, a mutation in 5-FCL gene doubled 5-FTHF levels and probably total folate levels in *arabidopsis* leaves (Goyer et al., 2005), implying the potential on enhance total folate levels. In this study, a marker for the function site of *ZmFCL* has been developed and could be applied to improve folate levels in sweet corn in the future.

## Conclusions

In this study, six folate derivatives were quantified in sweet corn kernels with three replications at 20 DAP. We performed a GWAS of 9.8 million SNPs to dissect the genetic architecture of folate related genes in sweet corn kernels. A total of 95 significant SNPs associated with eight folate traits were identified, and 73 candidate genes were nominated. The gene *ZmFCL* involved in the folate pathway was significantly associated with 5-FTHF content. Candidate gene association and haplotype analyses revealed that two adjacent SNPs on the second exon may be the causative variant. This study improves our understanding of the genetic basis of folate and provides molecular markers for folate biofortification in sweet corn kernels.

## Data availability statement

The raw genomic sequencing data and RNA sequencing data have been deposited into the China National GeneBank DataBase (CNGBdb) repository, accession numbers CNP0003213 and CNP0003294.

## Author contributions

JH and GL conceived the research and designed the experiments. YX and YY performed the experiments, population collection and wrote the manuscript. LX performed phenotypic determination. KL performed some of the data analysis. XG and GyL and JL performed the field experiments. All authors contributed to the article and approved the submitted version.

## Funding

This study was supported by the key area research and development program of Guangdong Province (2018B020202008), provincial rural revitalization strategy special project of Guangdong in 2022 (No. 92), construction and operation of the Food Nutrition and Health Research Center of Guangdong Academy of Agricultural Sciences (XTXM 202205), agricultural competitive industry discipline team building project of Guangdong Academy of Agricultural Sciences(202115TD), Special Fund for Scientific Innovation Strategy-Construction of High Level Academy of Agriculture Science (R2017YJ-YB1002).

## Conflict of interest

The authors declare that the research was conducted in the absence of any commercial or financial relationships that could be construed as a potential conflict of interest.

## Publisher’s note

All claims expressed in this article are solely those of the authors and do not necessarily represent those of their affiliated organizations, or those of the publisher, the editors and the reviewers. Any product that may be evaluated in this article, or claim that may be made by its manufacturer, is not guaranteed or endorsed by the publisher.

## Supplementary material

Supplementary Figure 1 The folate biosynthesis and C1-metabolism pathway in plant cells. The five folate derivatives detected in this study are shown with red text. Folic acid is a free acid and not shown in the pathway. The enzymes involved in the pathway are shown in italic text, including aminodeoxychorismate synthase (ADCS), aminodeoxychorismate lyase (ADCL), GTP cyclohydrolase I (GTPCHI), dihydroneopterin aldolase (DHNA), DHN-P3-diphosphatase, hydroxymethyldihydropterin pyrophosphokinase (HPPK), dihydropteroate synthase (DHPS), dihydrofolate synthetase (DHFS), dihydrofolate reductase (DHFR), 5,10-methylene-THF dehydrogenase/5,10-methenyl-THF cyclohydrolase (DHC), folylpolyglutamate synthetase (FPGS), serine hydroxymethyl transferase 1 (SHMT1), glycine decarboxylase complex (GDC), 5,10-methylenetetrahydrofolate reductase (MTHFR), 10-formyl THF deformylase (10-FDF), 10-formyltetrahydrofolate synthetase (FTHS), 5-formyl-THF cycloligase (5-FCL), folylpolyglutamate synthetase (FPGS) and glutamyl hydrolase (GGH). (modified with Ravanel et al., 2011).

Supplementary Figure 2 Manhattan and quantile-quantile Plots of eight folate traits in the association population. The red dashed horizontal line depicts the Bonferroni-adjusted significance threshold (*P* = 1.0 × 10^−6^). *ZmFCL*, significantly associated with 5-FTHF content, is highlighted with red.

Supplementary Figure 3 Functional enrichment analysis of candidate genes.

Supplementary Figure 4 Protein analysis and phylogenetic analysis of *ZmFCL*. **(A)**Protein model of *ZmFCL*. Blue and pink regions represent N-terminal and C-terminal subdomain superfamily, respectively. The substitution of amino acid translated by causative variation was highlighted with yellow circle. **(B)**The neighbor-joining phylogenetic tree of *ZmFCL* and its orthologs from major cereals and vegetables. Bootstrap values from 1,000 replicates are indicated at each node, and the scale represents branch length. ZmFCL (B73) and ZmFCL (RC) represented field corn and sweet corn, respectively.

Supplementary Figure 5 Amino acid sequence alignment of ZmFCL and its orthologs in various species. Zm0001d13786 (B73), Zm0001d13786 (RC), SORBI_3001G181300, SETIT_038737mg, Os03g0582000, BGIOSGA013019, TraesCS1B02G321700 and HORVU.MOREX.r3.1HG0069460 were from field corn, sweet corn, *Sorghum bicolor*, *Setaria italic*, *O. japonica*, *O*.*indica*, *Triticum aestivum*, and *Hordeum vulgare*, respectively. The numbers to the right indicate the amino acid sequence positions. Green helices represent α-helices, blue arrows indicate β-strands. The secondary structure was predicted by *Phyre2*. The 228^th^ amino acid in the protein was highlighted with red.
