# Supplementary figures and images for: A genome-wide association study of folates in sweet corn kernels
Source: Front Plant Sci. 2022 Sep 30;13:1004455. doi: 10.3389/fpls.2022.1004455 (PMC9562826; doi:10.3389/fpls.2022.1004455)

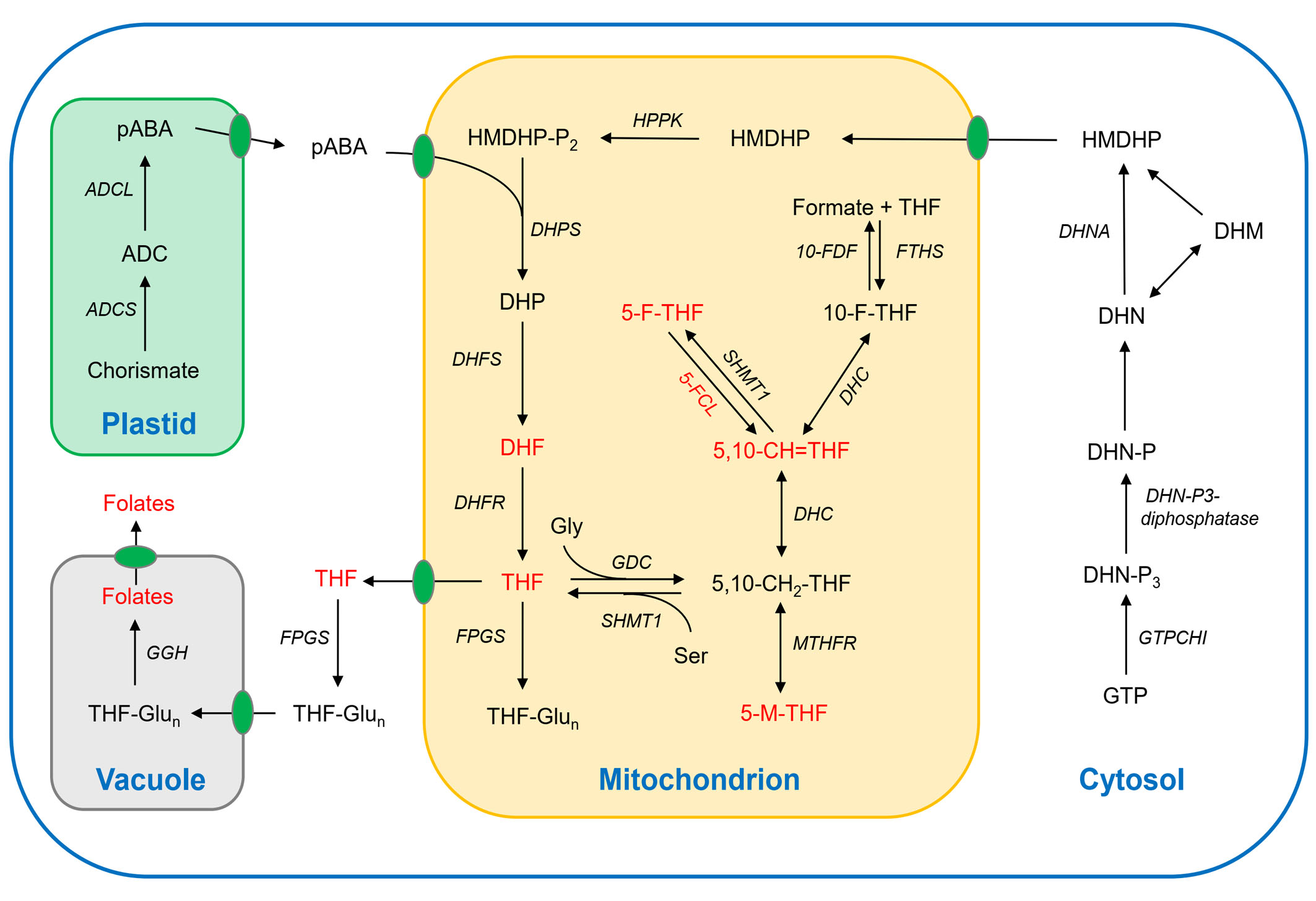

Supplement: Supplementary Figure 1 — The folate biosynthesis and C1-metabolism pathway in plant cells. The five folate derivatives detected in this study are shown with red text. Folic acid is a free acid and not shown in the pathway. The enzymes involved in the pathway are shown in italic text, including aminodeoxychorismate synthase (ADCS), aminodeoxychorismate lyase (ADCL), GTP cyclohydrolase I (GTPCHI), dihydroneopterin aldolase (DHNA), DHN-P3-diphosphatase, hydroxymethyldihydropterin pyrophosphokinase (HPPK), dihydropteroate synthase (DHPS), dihydrofolate synthetase (DHFS), dihydrofolate reductase (DHFR), 5,10-methylene-THF dehydrogenase/5,10-methenyl-THF cyclohydrolase (DHC), folylpolyglutamate synthetase (FPGS), serine hydroxymethyl transferase 1 (SHMT1), glycine decarboxylase complex (GDC), 5,10-methylenetetrahydrofolate reductase (MTHFR), 10-formyl THF deformylase (10-FDF), 10-formyltetrahydrofolate synthetase (FTHS), 5-formyl-THF cycloligase (5-FCL), folylpolyglutamate synthetase (FPGS) and glutamyl hydrolase (GGH). (modified with Ravanel et al., 2011). [file DataSheet_1.zip › Figure S1.JPEG]

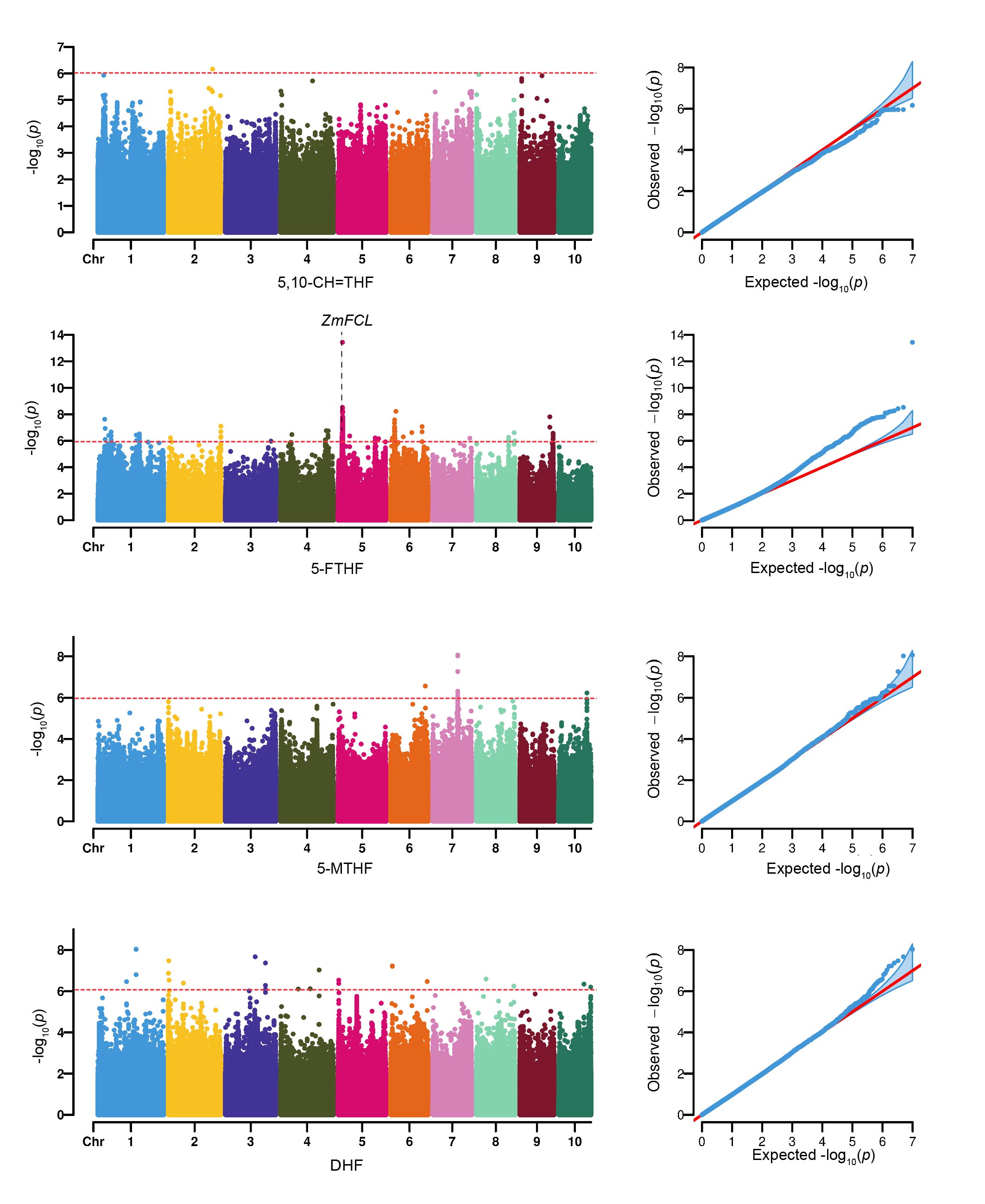

Supplement: Supplementary Figure 1 — The folate biosynthesis and C1-metabolism pathway in plant cells. The five folate derivatives detected in this study are shown with red text. Folic acid is a free acid and not shown in the pathway. The enzymes involved in the pathway are shown in italic text, including aminodeoxychorismate synthase (ADCS), aminodeoxychorismate lyase (ADCL), GTP cyclohydrolase I (GTPCHI), dihydroneopterin aldolase (DHNA), DHN-P3-diphosphatase, hydroxymethyldihydropterin pyrophosphokinase (HPPK), dihydropteroate synthase (DHPS), dihydrofolate synthetase (DHFS), dihydrofolate reductase (DHFR), 5,10-methylene-THF dehydrogenase/5,10-methenyl-THF cyclohydrolase (DHC), folylpolyglutamate synthetase (FPGS), serine hydroxymethyl transferase 1 (SHMT1), glycine decarboxylase complex (GDC), 5,10-methylenetetrahydrofolate reductase (MTHFR), 10-formyl THF deformylase (10-FDF), 10-formyltetrahydrofolate synthetase (FTHS), 5-formyl-THF cycloligase (5-FCL), folylpolyglutamate synthetase (FPGS) and glutamyl hydrolase (GGH). (modified with Ravanel et al., 2011). [file DataSheet_1.zip › Figure S2A.JPEG]

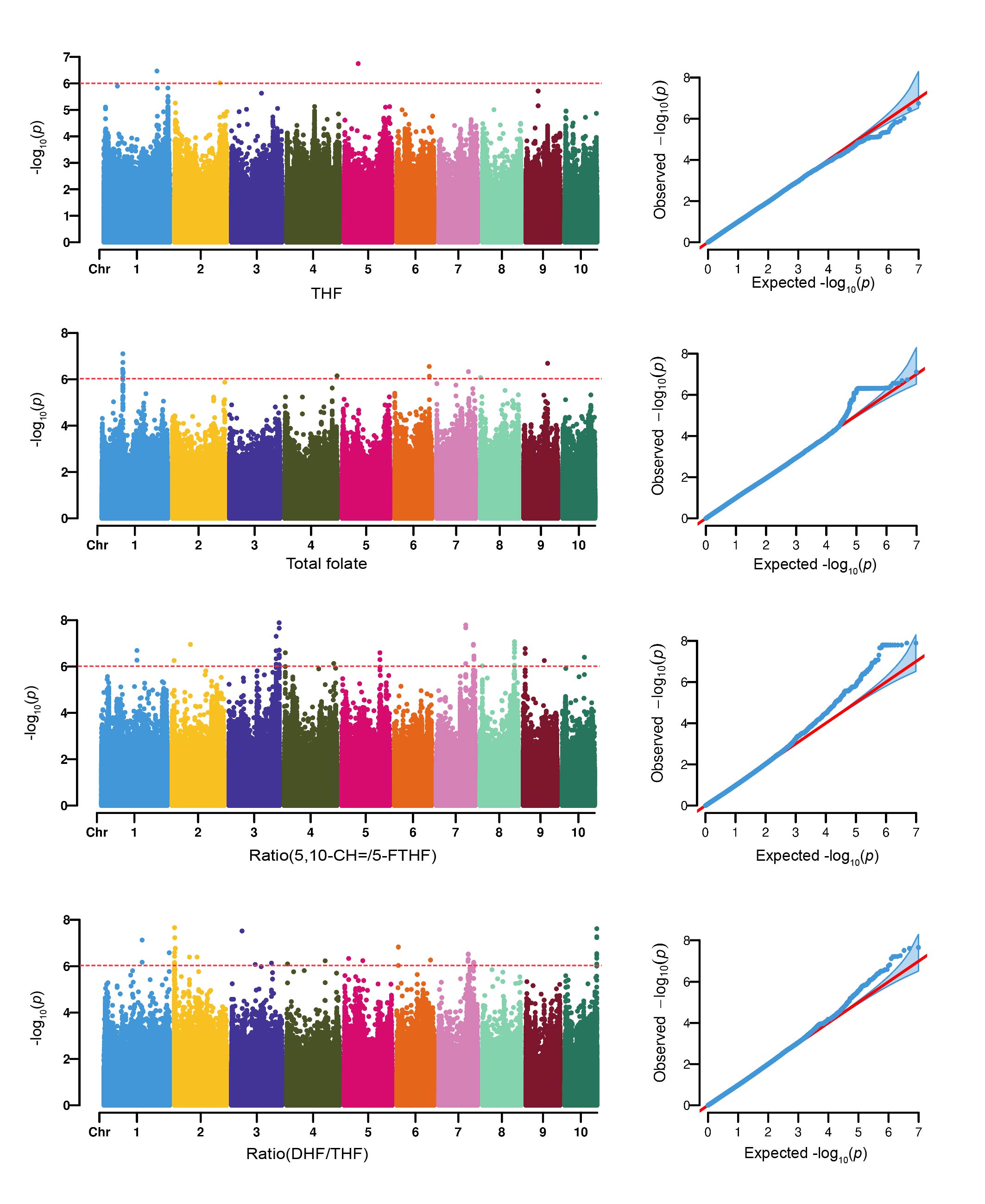

Supplement: Supplementary Figure 1 — The folate biosynthesis and C1-metabolism pathway in plant cells. The five folate derivatives detected in this study are shown with red text. Folic acid is a free acid and not shown in the pathway. The enzymes involved in the pathway are shown in italic text, including aminodeoxychorismate synthase (ADCS), aminodeoxychorismate lyase (ADCL), GTP cyclohydrolase I (GTPCHI), dihydroneopterin aldolase (DHNA), DHN-P3-diphosphatase, hydroxymethyldihydropterin pyrophosphokinase (HPPK), dihydropteroate synthase (DHPS), dihydrofolate synthetase (DHFS), dihydrofolate reductase (DHFR), 5,10-methylene-THF dehydrogenase/5,10-methenyl-THF cyclohydrolase (DHC), folylpolyglutamate synthetase (FPGS), serine hydroxymethyl transferase 1 (SHMT1), glycine decarboxylase complex (GDC), 5,10-methylenetetrahydrofolate reductase (MTHFR), 10-formyl THF deformylase (10-FDF), 10-formyltetrahydrofolate synthetase (FTHS), 5-formyl-THF cycloligase (5-FCL), folylpolyglutamate synthetase (FPGS) and glutamyl hydrolase (GGH). (modified with Ravanel et al., 2011). [file DataSheet_1.zip › Figure S2B.JPEG]

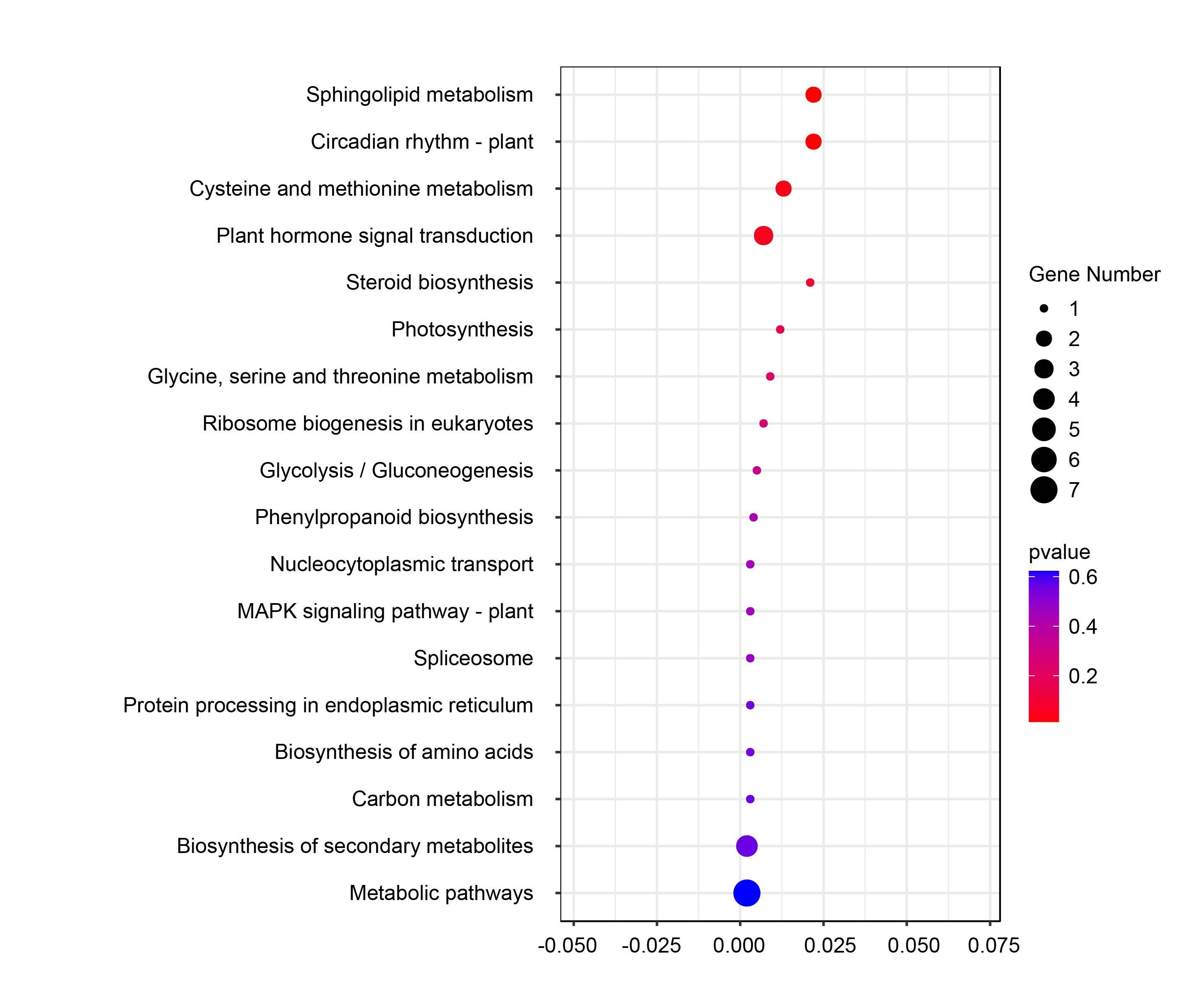

Supplement: Supplementary Figure 1 — The folate biosynthesis and C1-metabolism pathway in plant cells. The five folate derivatives detected in this study are shown with red text. Folic acid is a free acid and not shown in the pathway. The enzymes involved in the pathway are shown in italic text, including aminodeoxychorismate synthase (ADCS), aminodeoxychorismate lyase (ADCL), GTP cyclohydrolase I (GTPCHI), dihydroneopterin aldolase (DHNA), DHN-P3-diphosphatase, hydroxymethyldihydropterin pyrophosphokinase (HPPK), dihydropteroate synthase (DHPS), dihydrofolate synthetase (DHFS), dihydrofolate reductase (DHFR), 5,10-methylene-THF dehydrogenase/5,10-methenyl-THF cyclohydrolase (DHC), folylpolyglutamate synthetase (FPGS), serine hydroxymethyl transferase 1 (SHMT1), glycine decarboxylase complex (GDC), 5,10-methylenetetrahydrofolate reductase (MTHFR), 10-formyl THF deformylase (10-FDF), 10-formyltetrahydrofolate synthetase (FTHS), 5-formyl-THF cycloligase (5-FCL), folylpolyglutamate synthetase (FPGS) and glutamyl hydrolase (GGH). (modified with Ravanel et al., 2011). [file DataSheet_1.zip › Figure S3.JPEG]

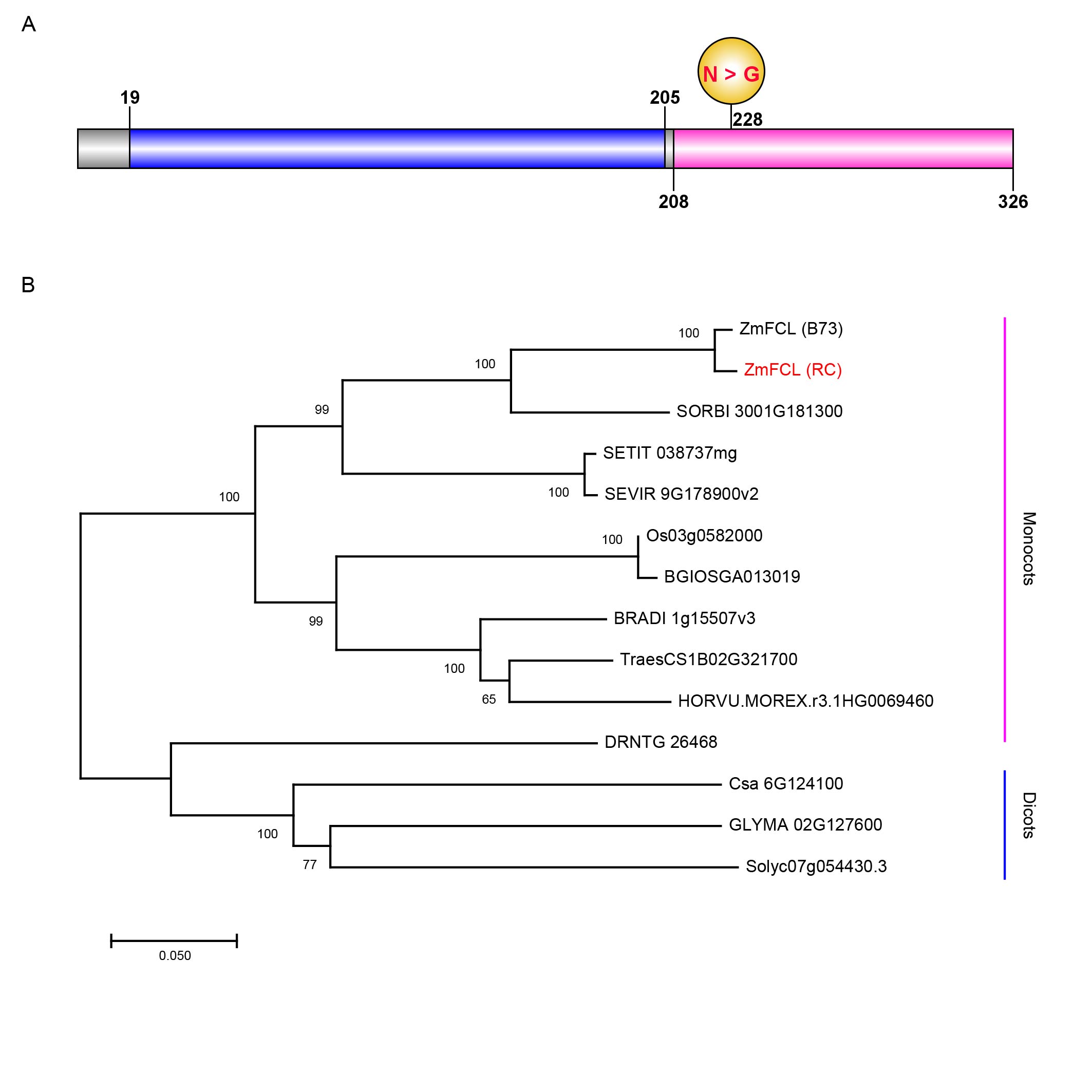

Supplement: Supplementary Figure 1 — The folate biosynthesis and C1-metabolism pathway in plant cells. The five folate derivatives detected in this study are shown with red text. Folic acid is a free acid and not shown in the pathway. The enzymes involved in the pathway are shown in italic text, including aminodeoxychorismate synthase (ADCS), aminodeoxychorismate lyase (ADCL), GTP cyclohydrolase I (GTPCHI), dihydroneopterin aldolase (DHNA), DHN-P3-diphosphatase, hydroxymethyldihydropterin pyrophosphokinase (HPPK), dihydropteroate synthase (DHPS), dihydrofolate synthetase (DHFS), dihydrofolate reductase (DHFR), 5,10-methylene-THF dehydrogenase/5,10-methenyl-THF cyclohydrolase (DHC), folylpolyglutamate synthetase (FPGS), serine hydroxymethyl transferase 1 (SHMT1), glycine decarboxylase complex (GDC), 5,10-methylenetetrahydrofolate reductase (MTHFR), 10-formyl THF deformylase (10-FDF), 10-formyltetrahydrofolate synthetase (FTHS), 5-formyl-THF cycloligase (5-FCL), folylpolyglutamate synthetase (FPGS) and glutamyl hydrolase (GGH). (modified with Ravanel et al., 2011). [file DataSheet_1.zip › Figure S4.JPEG]

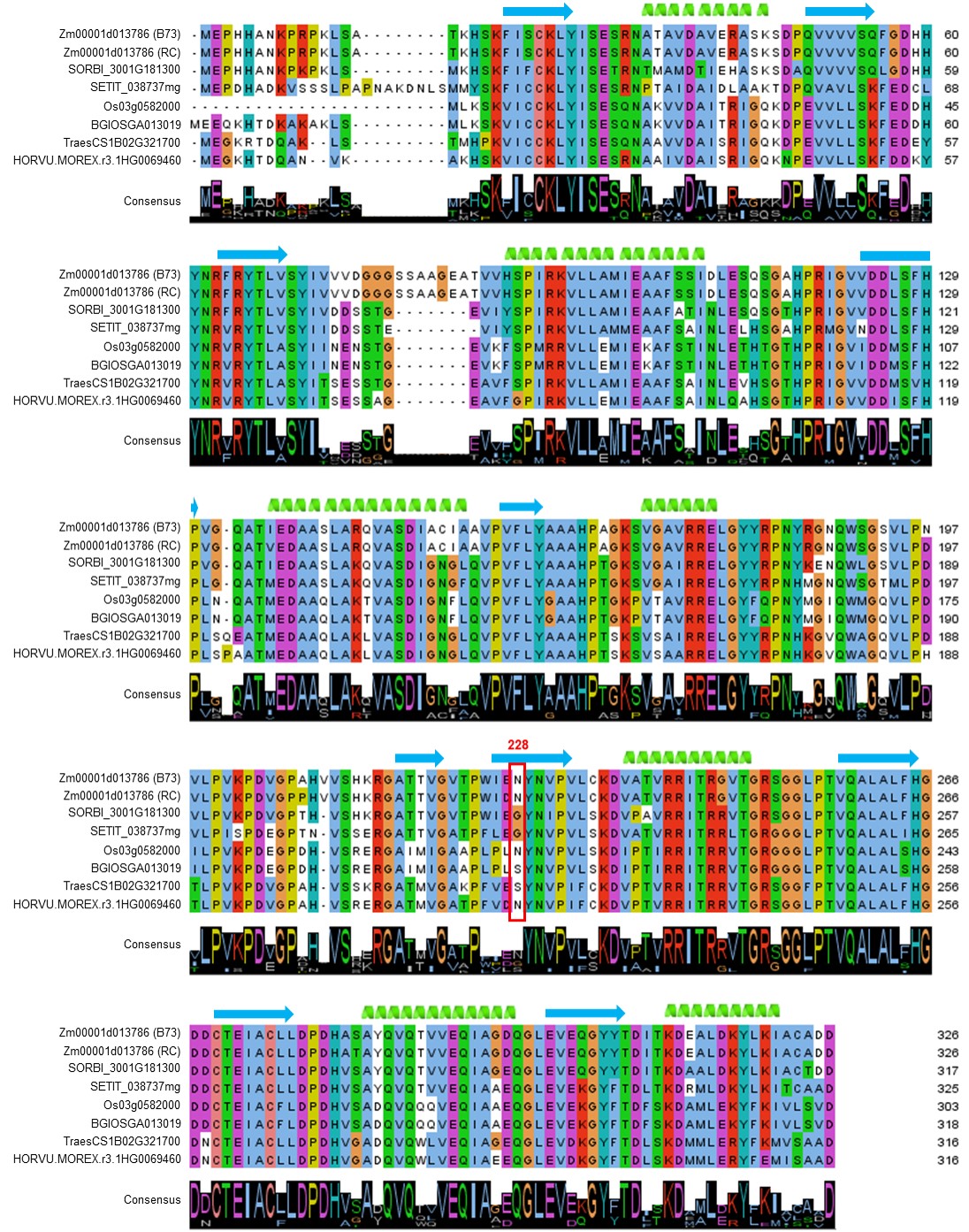

Supplement: Supplementary Figure 1 — The folate biosynthesis and C1-metabolism pathway in plant cells. The five folate derivatives detected in this study are shown with red text. Folic acid is a free acid and not shown in the pathway. The enzymes involved in the pathway are shown in italic text, including aminodeoxychorismate synthase (ADCS), aminodeoxychorismate lyase (ADCL), GTP cyclohydrolase I (GTPCHI), dihydroneopterin aldolase (DHNA), DHN-P3-diphosphatase, hydroxymethyldihydropterin pyrophosphokinase (HPPK), dihydropteroate synthase (DHPS), dihydrofolate synthetase (DHFS), dihydrofolate reductase (DHFR), 5,10-methylene-THF dehydrogenase/5,10-methenyl-THF cyclohydrolase (DHC), folylpolyglutamate synthetase (FPGS), serine hydroxymethyl transferase 1 (SHMT1), glycine decarboxylase complex (GDC), 5,10-methylenetetrahydrofolate reductase (MTHFR), 10-formyl THF deformylase (10-FDF), 10-formyltetrahydrofolate synthetase (FTHS), 5-formyl-THF cycloligase (5-FCL), folylpolyglutamate synthetase (FPGS) and glutamyl hydrolase (GGH). (modified with Ravanel et al., 2011). [file DataSheet_1.zip › Figure S5.JPEG]
